# Vitamin D deficiency and vitamin D receptor FokI polymorphism as risk factors for COVID-19

**DOI:** 10.1038/s41390-022-02275-6

**Published:** 2022-09-09

**Authors:** Nancy M. S. Zeidan, Hanan M. Abd El Lateef, Dalia M. Selim, Suzan A. Razek, Ghada A. B. Abd-Elrehim, Mohamed Nashat, Noha ElGyar, Nevin M. Waked, Attia A. Soliman, Ahmed A. Elhewala, Mohamed M. M. Shehab, Ahmed A. A. Ibraheem, Hassan Shehata, Yousif M. Yousif, Nagwa E. Akeel, Mustafa I. A. Hashem, Amani A. Ahmed, Ahmed A. Emam, Mohamed M. Abdelmohsen, Mohamed F. Ahmed, Ahmed S. E. Saleh, Heba H. Eltrawy, Gehan H. Shahin, Rehab M. Nabil, Thoraya A. Hosny, Mohamed R. Abdelhamed, Mona R. Afify, Mohanned T. Alharbi, Mohammed K. Nagshabandi, Muyassar K. Tarabulsi, Sherif F. Osman, Amal S. M. Abd-Elrazek, Manal M. Rashad, Sonya A. A. El-Gaaly, Said A. B. Gad, Mohamed Y. Mohamed, Khalil Abdelkhalek, Aly A. Yousef

**Affiliations:** 1 Department of Pediatrics, Faculty of Medicine, Cairo University, Giza, Egypt; 2 Department of Pediatrics, Faculty of Medicine, Ain-Shams University, Cairo, Egypt; 3 Department of Pediatrics, Faculty of Medicine, Sohag University, Sohag, Egypt; 4 Department of Pediatrics, Faculty of Medicine, Aswan University, Aswan, Egypt; 5 Department of Pediatrics, Faculty of Medicine, Assuit University, Assuit, Egypt; 6 Department of Pediatrics, Faculty of Medicine, October 6 University, 6th of October City, Egypt; 7 Department of Pediatrics, Faculty of Medicine, Zagazig University, Zagazig, Egypt; 8 Department of Pediatrics, Faculty of Medicine for Boys, Al-Azhar University, Cairo, Egypt; 9 Department of Otorhinolaryngology, Faculty of Medicine, Benha University, Benha, Egypt; 10 Department of Chest Diseases, Faculty of Medicine for Girls, Al-Azhar University, Cairo, Egypt; 11 Department of Clinical Pathology, Faculty of Medicine, Cairo University, Giza, Egypt; 12 Department of Clinical Pathology, Faculty of Medicine, Zagazig University, Zagazig, Egypt; 13 Department of Clinical Pathology, Al-Azhar Faculty of Medicine, Cairo, Egypt; 14 Department of Medical Microbiology and Parasitology, Faculty of Medicine, University of Jeddah, Jeddah, Saudi Arabia; 15 Department of Radiology, Texas Tech University Health Sciences Center, El Paso, TX USA; 16 Department of Radio-Diagnosis, Menoufia University, Shibin Al Kawm, Egypt; 17 Department of Anesthesia, Faculty of Medicine, Zagazig University, Zagazig, Egypt; 18 Department of Internal Medicine, Faculty of Medicine, Ain-Shams University, Cairo, Egypt; 19 Department of Internal Medicine, Faculty of Medicine, Zagazig University, Zagazig, Egypt; 20 Department of Psychiatry, Faculty of Medicine, Ain-Shams University, Cairo, Egypt; 21 Department of Pediatrics, Faculty of Medicine, Helwan University, Helwan, Egypt

## Abstract

**Background:**

Given the sparse data on vitamin D status in pediatric COVID-19, we investigated whether vitamin D deficiency could be a risk factor for susceptibility to COVID-19 in Egyptian children and adolescents. We also investigated whether vitamin D receptor (VDR) FokI polymorphism could be a genetic marker for COVID-19 susceptibility.

**Methods:**

One hundred and eighty patients diagnosed to have COVID‐19 and 200 matched control children and adolescents were recruited. Patients were laboratory confirmed as SARS-CoV-2 positive by real-time RT-PCR. All participants were genotyped for VDR Fok1 polymorphism by RT-PCR. Vitamin D status was defined as sufficient for serum 25(OH) D at least 30 ng/mL, insufficient at 21–29 ng/mL, deficient at <20 ng/mL.

**Results:**

Ninety-four patients (52%) had low vitamin D levels with 74 (41%) being deficient and 20 (11%) had vitamin D insufficiency. Vitamin D deficiency was associated with 2.6-fold increased risk for COVID-19 (OR = 2.6; [95% CI 1.96–4.9]; *P* = 0.002. The FokI FF genotype was significantly more represented in patients compared to control group (OR = 4.05; [95% CI: 1.95–8.55]; *P* < 0.001).

**Conclusions:**

Vitamin D deficiency and VDR Fok I polymorphism may constitute independent risk factors for susceptibility to COVID-19 in Egyptian children and adolescents.

**Impact:**

- Vitamin D deficiency could be a modifiable risk factor for COVID-19 in children and adolescents because of its immune-modulatory action.
- To our knowledge, ours is the first such study to investigate the VDR *Fok* I polymorphism in Caucasian children and adolescents with COVID-19.
- Vitamin D deficiency and the VDR *Fok* I polymorphism may constitute independent risk factors for susceptibility to COVID-19 in Egyptian children and adolescents.
- Clinical trials should be urgently conducted to test for causality and to evaluate the efficacy of vitamin D supplementation for prophylaxis and treatment of COVID-19 taking into account the VDR polymorphisms.

## Introduction

In late December 2019, an outbreak of pneumonia was initially reported in Wuhan, China and later named the novel coronavirus disease 2019 (COVID-19) caused by severe acute respiratory syndrome coronavirus 2 (SARS-CoV-2).^1^ The World Health Organization (WHO) has announced it as a global healthcare pandemic on March 2020. Since then, more than 519 million cases and about 6 million deaths have been reported worldwide.^2^ SARS-CoV-2 enters the host cells via binding to the angiotensin-converting enzyme-2 (ACE-2) receptors, mainly expressed on alveolar Type‐II pneumocytes, vascular endothelial cells, epithelial cells in the kidney, and enterocytes of the small intestine.^3^ Children with COVID‐19 may be asymptomatic or with mild clinical course as compared to SARS-CoV-2-infected adults and reports of death are scarce.^4^ However, pneumonia followed by acute respiratory distress syndrome (ARDS), sepsis, and multiple organ failure are serious complications of COVID-19.^5^

Vitamin D deficiency represents a major public health issue since over one billion people are estimated to have vitamin D deficiency worldwide. Vitamin D is a fat-soluble secosteroid pro-hormone essential for bone metabolism and mineral homeostasis. Calcitriol [1,25-dihydroxyvitamin D3], an activated analog of vitamin D3, exerts its biological functions through vitamin D receptors (VDRs) mainly expressed on the gut, bone, lungs, and the majority of immune cells.^6^ Although the VDR is highly expressed in lung tissue, the potential role of vitamin D-VDR signaling in pulmonary immunopathology remains to be defined.

A meta-analysis of 25 randomized controlled trials confirmed that frequent vitamin D supplementation generally protects against acute lower respiratory infections and its deficiency is a risk factor for pneumonia and ARDS.^7,8^

Vitamin D not only enhances innate immune response but also modulates adaptive immunity and regulates the inflammatory cascade.^7,9^ Vitamin D enhances the expression of VDRs and recognition of viral dsRNA by Toll like receptor 3. It also induces secretion of antiviral peptides such as cathelicidin and beta-defensin-2 peptides that can block viral entry into cells and suppress viral replication rate.^10^ Moreover, it suppresses the expression of pro-inflammatory cytokines by CD4^+^ T cells that contribute to the cytokine storm, a major driver of illness severity in COVID-19.^11^

A recent study in the mainland of United States reported that sunlight exposure and adequate vitamin D status, with latitude as an indicator, was associated with reduced risk for COVID-19 and related complications.^12^ The highest age-specific case fatality rate of COVID-19 was estimated in Italy, Spain, and France where severe vitamin D deficiency is more prevalent than other European countries.^13^

The human VDR gene maps to chromosome 12q13. More than 470 single-nucleotide polymorphisms (SNPs) have been identified. However, only few of them modulate vitamin D uptake. Four major SNPs of VDR gene have been shown to influence VDR mRNA stability, expression, and activity [e.g., BsmI, TaqI, ApaI, and FokI].^14^

Among the VDR polymorphisms, FokI SNP is located at the translation start codon that results in transcription of two different VDR proteins, i.e., a short variant (F-VDR) or a longer form (f-VDR).^15^ A unique role of FokI polymorphism has been reported as the short F-VDR was found to influence immune cells’ behavior and always correlated with a more active immune system.^16^

To date, only a few studies in medical English literature reported that vitamin D deficiency may be associated with increased susceptibility to COVID‐19 disease in children and adolescents.^17–19^

Given the sparse data on vitamin D status in pediatric COVID-19 cases, we investigated whether vitamin D deficiency could be a risk factor for susceptibility to COVID-19 and the severity of illness in Egyptian children and adolescents. We also investigated whether the VDR Fok1 (rs2228570, *C/T*) polymorphism could be a genetic marker for COVID-19 susceptibility.

## Methods

This prospective multicenter study was performed at Cairo, Ain-Shams, and Assuit University hospitals from October 2020 through March 2021. The study protocol was approved by medical Ethics committees at Cairo, Ain-Shams, and Assuit Universities, Egypt. The study was conducted in accordance with the Declaration of Helsinki and written informed parental consent was obtained for all participants.

### Case definition

One hundred and eighty patients aged <19 years who were diagnosed to have COVID‐19 at the study hospitals were recruited. All patients were laboratory confirmed as SARS-CoV-2 positive by real-time reverse transcriptase polymerase chain reaction (RT-PCR) assay of nasopharyngeal swab specimens.

Patients’ COVID-19 illness severity was categorized into moderate, severe, and critical subgroups according to the recently published classification by Chen et al.^20^

No asymptomatic or mild cases were seen among our cohort.

(I) Moderate: included (134) cases presented with pneumonia (lower respiratory symptoms plus fever >38 °C and age-specific tachypnea);

(II) Severe COVID-19: included (31) cases who rapidly develop dyspnea, hypoxia (arterial oxygen saturation <93%), dehydration with feeding difficulty, elevated liver enzymes, disturbed consciousness, coagulation dysfunction;

(III) Critically ill cases: included (15) patients who required intensive care unit (ICU) monitoring for acute respiratory failure (ARF), mechanical ventilation (partial arterial oxygen pressure/fraction of inspired oxygen [PaO_2_/FiO_2_] ratio ≤300 despite oxygen therapy), septic shock, or organ failure.

Patients were admitted within 72 h from onset of fever and cough. Pulmonary high-resolution computed tomographic images were routinely performed for all patients and evaluated by two experienced radiologists (S.F.O. and A.S.M.) who were blinded to the patients’ clinical data.

### Control group

Two hundred healthy children and adolescents of matched age, sex, and season at enrollment who underwent pre-operative assessment for elective surgery at the study hospitals were enrolled as a control group (all tested negative for SARS-Cov2 by RT-PCR and had negative anti-N antibodies test for SARS-Cov2).

All patients and control subjects belong to the same ethnic group: African Caucasian.

### Exclusion criteria

Patients with obesity, malnutrition, immunodeficiency, congenital heart disease, malignancy, metabolic diseases, autoimmune disorders, or any chronic debilitating disease were excluded. Those who received vitamin D, calcium, multi-vitamin, or mineral supplementation during the previous 6 months were also excluded.

### Laboratory investigations

Upon enrollment, 5 mL venous blood sample was drawn for molecular and serological evaluation. Laboratory biomarkers including complete blood count, C‐reactive protein (CRP), lactate dehydrogenase, serum ferritin, and D-dimer were evaluated. Serum parathyroid hormone was measured using ElectroChemiLuminscence immunoassay (Roche Diagnostics GmbH, Germany).

### Estimation of serum [25(OH)] D levels

Serum 25(OH) D concentrations was measured using ELISA Kit (K2110 Immunodiagnostic [Dutch Company], Holland) according to the manufacturer’s instructions.

Vitamin D status was defined as sufficient for those having serum 25(OH) D level at least 30 ng/mL (75 nmol/L), insufficient at 21–29 ng/mL (52.5 and 72.5 nmol/l), deficient at <20 ng/mL(<50 nmol/l), and <10 ng/ml (25 nmol/L) as severe deficiency.^21^

Patients diagnosed with COVID‐19 were further subdivided into two groups. Those with serum 25(OH) D level <30 ng/ml (75 nmol/L) were determined as Group 1 (vitamin D insufficient and deficient) and those with 25(OH) D levels ≥30 ng/mL as Group 2 (normal VD status).

### RNA extraction and SARS-CoV-2 diagnosis

Nasopharyngeal swabs from all subjects were collected on 0.5 mL TE pH 8 buffer. Viral RNA was automatically extracted using magnetic beads on Chemagic 360 (Perkin Elmer, Germany). Detection of SARS-CoV-2 was done by real-time PCR NAT using Viasure SARS-CoV-2 RT-qPCR kit (CerTest Biotec; Spain) on CFX96 BioRad as described by Freire-Paspuel et al.^22^ The Viasure SARS-COV2 detection kit had 97.5% sensitivity and 100% specificity compared to CDC FDA EUA kit as gold standard.

### Genotyping for VDR Fok1 polymorphism

Genomic DNA extraction was performed from 200 μL whole-blood sample using the QIAamp Blood mini kit (Qiagen, Hilden, Germany) and stored at −20 °C before genotyping. All participants were genotyped for the VDR Fok1 (rs2228570, 27823 *C/T*) polymorphism by Real Time (RT-PCR) using TaqMan® Universal PCR Master Mix (Applied Biosystems, PN 4304437). The rs2228570/*Fok*1 (*C/T*) SNP in exon 2 was evaluated by allelic discrimination RT-PCR using TaqMan® probes (Applied Biosystems, Foster City, Canada). The following primers were used: Short primer 5′CGCACAGACAGGCCTGCA-3′, Long primer 5′-TGCCGCCTGCCTGCGCAGACAGGCCTGCG-3′, and Constant primer 5′-GCCCAGTTCACGCAAGAG-3′ as previously described by Fedirko et al.^23^

### Statistical analysis

Statistical analysis was performed by the SPSS software for windows, version 20 (IBM, Chicago). Continuous parameters were compared using Student’s *t* test, analysis of variance test, or Mann–Whitney *U*-test, as appropriate. Association of categorical variables, genotype distribution, and allele frequencies were compared using the Chi-square (*χ*^2^) test after calculation of the Hardy–Weinberg equilibrium (HWE). Logistic regression analyses were applied to calculate odds ratios with 95% confidence intervals [OR; 95% CIs] for different VDR genotypes and to quantify the independent effect of serum 25(OH) D levels and VDR *Fok* I genotypes on disease susceptibility.

A *P* value of <0.05 was considered statistically significant.

## Results

Over the 6-month study period, 180 patients with laboratory-confirmed COVID-19 and 200 healthy control subjects were enrolled. The median age of patients was 10.7 years, range (8.3–18.9) years, and 110 (61%) were males. The control group were well matched for age, sex, and season at enrollment [median age 11.4 years; range (8.5–19) years] and 126 (63%) were males (*P* = 0.721, *P* = 0.352, respectively); Table 1.

**Table 1:** The demographic data and laboratory characteristics of COVID-19 patients and control group.

	Patients (*n* = 180)	Control group (*n* = 200)	*P*
Age (years)	10.7 (8.3–18.9)	11.4 (8.5–19)	0.721
Male, *n* (%)	110 (61)	126 (63)	0.352
Season at enrollment (winter/non-winter)	144 (80)/36 (20)	162 (81)/38 (19)	0.518
Family member with COVID-19	142 (78.8)	—	—
Vitamin D level (ng/mL)	14.7 (6.4–53)	37.6 (10.3–72.8)	<0.001
Parathyroid hormone level (pg/mL)	46.4 (32.5–81.6)	42.8 (28.3–76.4)	0.382
Serum calcium (mg/dL)	8.8 ± 0.7	10.3 ± 0.5	0.018
Serum phosphorus (U/L)	4.2 ± 0.68	4.9 ± 0.76	0.023
Serum alkaline phosphatase (mg/dL)	212 ± 59	203 ± 66	0.435

Values in parentheses are percentages or data are presented as mean ± SD or median (range).

*COVID-19* coronavirus disease 2019.

*P* value < 0.05 indicates a significant difference.

In all, 78.8% of patients had a definitive contact history with a family member with COVID-19. The baseline demographic and laboratory variables of patients and control group are listed in Table 1.

COVID-19 patients had significantly lower median serum 25-(OH) D levels 14.7 ng/mL (range; 6.4–53) than did the control group 37.6 ng/mL (10.3–72.8); *P* < 0.001, Table 1. Serum calcium and phosphorus levels were also significantly lower in patients compared to controls (serum Ca; 8.8 ± 0.7 vs 10.3 ± 0.5 mg/dL; *P* = 0.018 and serum P; 4.2 ± 0.68 vs 4.9 ± 0.76 U/L); *P* = 0.023, respectively, Table 1.

Fever, dry cough, and polypnea were the most prevalent symptoms on admission. Fifty-six (31%) patients developed pneumonia and 43 (23.8%) patients had dyspnea; Table 2. Of all the patients, 134 (74.4%) had moderate clinical type; 30 (16.7%) cases had severe COVID-19, and 16 (8.9%) were critical cases. Sixteen patients were admitted to pediatric ICU (PICU): 12 (6.7%) cases required mechanical ventilation for ARF and 4 patients (2.2 %) had septic shock; Table 2. All patients survived.

**Table 2:** Comparison of clinical and laboratory variables between COVID-19 patient subgroups.

	Group 1 vitamin D deficient (*n* = 94)	Group 2 normal vitamin D (*n* = 86)	*P*
Age (years)	10.5 (8.3–18.6)	10.7 (8.5–18.9)	0.676
Male, *n* (%), 110	59 (61)	51 (60.7)	0.432
COVID-19 severity			
Moderate, *n* = 134	68 (72.3)	66 (76.7)	
Severe, *n* = 30	17 (18.08)	13 (15)	0.795
Critical, *n* = 16	9 (9.6)	7 (8.1)	
Fever >38 °C, *n* = 157	84 (89.3)	73 (84.8)	0.253
Dry cough, *n* = 102	57 (60.6)	45 (52)	0.087
Dyspnea, *n* = 43	23 (24)	20 (23)	0.546
Nausea/vomiting, *n* = 29	16 (17)	13 (15)	0.883
Diarrhea, *n* = 38	21 (22)	17 (19.7)	0.167
Fatigue, *n* = 22	12 (12.7)	10 (11.6)	0.839
Hypoxemia, *n* = 34	19 (20)	15 (17.4)	0.321
Cyanosis, *n* = 19	11 (11.7)	8 (9.3)	0.476
Pneumonia, *n* = 56	32 (34)	24 (28)	0.059
ARF, *n* = 12	7 (7.4)	5 (5.8)	0.221
Shock, *n* = 4	2 (2.1)	2 (2.3)	0.877
Duration of fever (in days)	7 (2–11)	5 (2–9)	0.056
Duration of hospital stay (in days)	6 (3–14)	5 (2–10)	0.358
Chest CT scan			
Ground-glass opacities, *n* = 56	32 (34)	24 (28)	0.668
Pulmonary consolidation, *n* = 48	27 (29)	21 (24)	0.723
Laboratory findings			
CRP (<8 mg/dL)	7.3 (0.98–25)	7.1 (0.88–23)	0.267
Vitamin D level (ng/mL)	11.65(5.73–17.86)	25.73 (21.8–58.6)	<0.001
Parathyroid hormone level (pg/mL)	47.6 (18.5–108.7)	43.4(22.65–81.6)	0.264
Serum calcium (mg/dL)	9.58 ± 0.83	10.23 ± 0.68	0.073
Serum phosphorus (U/L)	3.87 ± 0.75	4.94 ± 0.83	0.026
Serum alkaline phosphatase (mg/dL)	217 ± 43	198 ± 56	0.143
Procalcitonin (<0.5 ng/mL)	13.2 (1.2‐18.7)	12.8 (1.1‐17.8)	0.154
D-dimer, (<0.5 μg/mL)	0.27 ± 1.3	0.24 ± 0.35	0.085
Lactate dehydrogenase (LDH) U/L	245 (200–307)	227 (198–304)	0.372
Serum Ferritin, ref. (15–140) ng/mL	41 (26–385)	38 (21–356)	0.343
White blood cell, ref. (4–10) × 10^9^/L	7.2 (5.6–11.6)	7.3 (5.5–11.4)	0.216
Neutrophils, ref. (2.0–7.2) × 10^9^/L	4.19 (2.34–7.54)	4.38 (2.46–7.68)	0.945
Lymphocytes, ref. (1.1–3.2) × 10^9^/L	2.18 (1.15–3.26)	2.35 (1.8–3.4)	0.058
Platelets, ref. (140–440) × 10^9^/L	167 (141–315)	187 (165–320)	0.132
ALT (<50 U/L)	21.5 (14–78)	19.6 (10.8–73)	0.085
AST (<60 U/L)	29 (14–95)	25 (13–89)	0.176
Creatinine (<62 μmol/L)	46.7 (32–78)	42 (29–58)	0.659

Values in parentheses are percentages or data are presented as mean ± SD or median (range).

*COVID-19* coronavirus disease 2019, *ARF* acute respiratory failure, *CRP* C‐reactive protein, *ALT* alanine aminotransferase, *AST* aspartate aminotransferase.

*P* value < 0.05 indicates a significant difference.

**Fig. 1:**
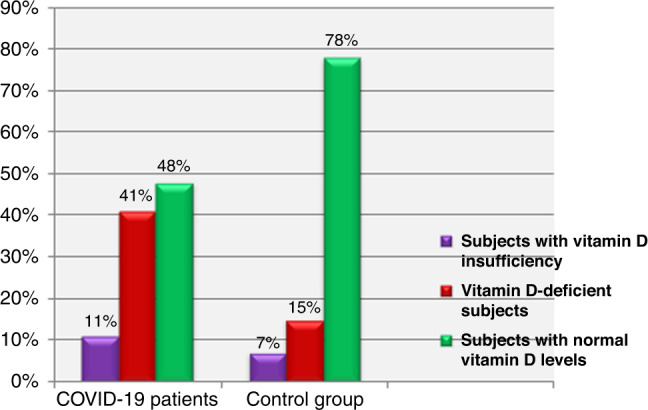
Vitamin D status among the studied subjects (*P* < 0.001).

Our data showed that 94 (52%) of COVID-19 patients had low vitamin D levels with 74 (41%) being deficient and 20 (11%) had vitamin D insufficiency; meanwhile, 86 (48%) patients had normal vitamin D level. By contrast, 30 (15%) of the control group were vitamin D deficient and 14 (7%) had vitamin D insufficiency; meanwhile, 156 (78%) had normal serum vitamin D level (*P* < 0.01; Fig. 1).

We next compared clinical data and laboratory parameters between COVID‐19-diagnosed patients who had deficient and insufficient vitamin D levels (Group 1; *n* = 94) and patients who had normal vitamin D levels (Group 2; *n* = 86) as presented in Table 2. There were significantly lower levels of [25(OH) D] and serum phosphorus in Group 1 than those in Group 2 (*P* < 0.001 and *P* = 0.026, respectively); Table 2. No significant difference was found between both groups as regards COVID-19 clinical presentation or disease severity as well as inflammatory biomarkers and other measured laboratory variables (all *P* > 0.05); Table 2.

The VDR FokI genotype distribution and allele frequencies for COVID-19 patients and control groups are presented in Table 3 and were compatible with the HWE.

**Table 3:** Distribution of the VDR *Fok* I genotypes, alleles, and serum 25-(OH) D in patients with COVID-19 and control group.

Genotype		Patient group	Control group	OR (95% CI)	*P*
		*n* (180), %	*n* (200), %		
VDR Fok I	*f/f*	83 (46)	96 (48)	Referent	
	*F/f*	60 (33)	92 (46)	0.59 (0.38–0.91)	0.01
	*F/F*	37 (21)	12 (6)	4.05 (1.95–8.55)	<0.001
Alleles	*f*	226 (62.8)	284 (71)	Referent	
	*F*	134 (37.2)	116 (29)	1.45 (1.06–1.99)	0.01
Serum 25(OH) D (ng/mL)		14.7 (6.4–53)	37.6 (10.3–72.8)		<0.01^a^

Values in parentheses are percentages or data are presented as mean ± SD.

*VDR* vitamin D receptor, *OR* odds ratio, *CI* 95% confidence interval.

*P* value < 0.05 indicates a significant difference. Chi-square test.

^a^Student’s *t* test.

The FokI homozygous *FF* genotype was significantly more represented in COVID-19 patients compared to the control group (21 vs 6%; OR = 4.05; [95% CI: 1.95–8.55]; *P* < 0.001); Table 3. Besides, the FokI *F* allele was more frequent among patients than healthy controls (37.2 vs 29%; OR = 1.45; [95% CI: 1.06–1.99]; *P* = 0.01); Table 3. Conversely, the *Fok* I *Ff* genotype was significantly under-represented in patients with COVID-19 compared to the control group (33 vs 46%; OR = 0.59; [95% CI: 0.38–0.91]; *P* = 0.01); Table 3. However, no significant associations was found between the VDR FokI genotype distribution and disease severity or clinical outcome (all *P* > 0.05); Table 4.

**Table 4:** Association of the VDR *Fok* I genotypes with disease severity and serum 25-hydroxyvitamin D in patients with COVID-19.

VDR *Fok* I genotype	*ff* (*n* = 83)	*Ff* (*n* = 60)	*FF* (*n* = 37)	*P*
	*n* (%)	*n* (%)	*n* (%)	
Severity				
Moderate (*n* = 134)	61 (73.4)	46 (77)	27 (73)	0.888
Severe (*n* = 30)	15 (18.2)	9 (15)	6 (16)	0.885
Critical (*n* = 16)	7 (8.4)	5 (8.3)	4 (11)	0.899^a^
ICU admission	7 (8.4)	5 (8.3)	4 (11	0.899^a^
Acute respiratory failure	5 (6)	4 (6.7)	3 (8)	0.914
Septic shock	2 (2.4)	1 (1.6)	1 (2.7)	0.933
Serum 25(OH) vitamin D (ng/mL)	23.5 ± 4.7	20.7 ± 6.5	^c^13.8 ± 5.6	<0.01^b^

*COVID-19* coronavirus disease 2019, *VDR* vitamin D receptor, *ICU* intensive care unit.

*P* value < 0.05 indicates a significant difference. Chi-square test.

^a^Fisher’s exact test.

^b^ANOVA test.

^c^Significant difference between each three genotype group.

**Fig. 2:**
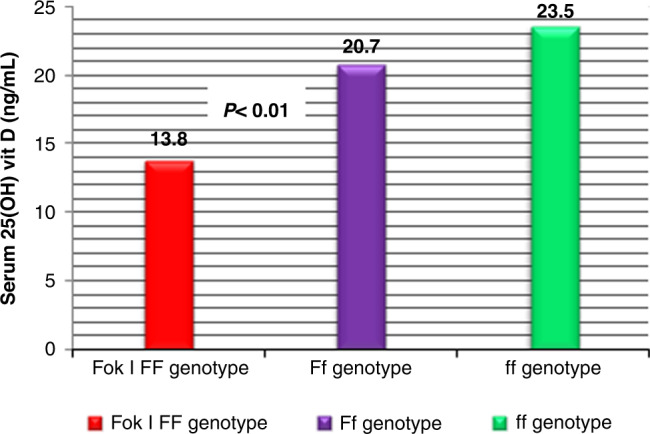
Mean serum 25(OH) D among the studied patients’ VDR Fok I genotypes.

Patients carrying the VDR *FF* genotype had lower [25(OH) D] serum levels (mean; 13.8 ± 5.6 ng/mL) as compared to those with *Ff* genotype (20.7 ± 6.5 ng/mL) and the *ff* genotype (23.5 ± 4.7 ng/mL); *P* < 0.01, respectively; Fig. 2.

Logistic regression analysis revealed that the VDR *Fok* I FF genotype and *F* allele were independent risk factors for COVID-19 among the studied patients (adjusted OR: 4.3; [95% CI: 2.6–9.2]; *P* < 0.001, for the *Fok* I FF genotype and adjusted OR: 2.25; [95% CI: 1.1–3.7]; *P* = 0.003 for the *F* allele) controlling for age, sex, season at enrollment, and household crowding; Table 5. Vitamin D deficiency was associated with 2.6-fold increased risk for COVID-19 (adjusted OR = 2.6; [95% CI 1.96–4.9]; *P* = 0.002; Table 5).

**Table 5:** Risk factors for susceptibility to COVID-19 among the studied patients.

Covariate	Univariate analysis		Multivariate analysis	
	Unadjusted OR (95% CI)	*P*	^a^Adjusted OR (95% CI)	*P*
Age	1.3 (0.8–2.7)	0.24		
Male	0.7 (0.61–1.9)	0.18		
Season at enrollment	1.2 (0.89–2.4)	0.31		
Weight for age *z*-scores	0.95 (0.57–1.6)	0.54		
Crowding (≥7 persons in household)	2.7 (0.6–17)	0.06		
Passive smoking	1.53 (0.71–3.30)	0.87		
Low parental education	1.01 (0.6–1.47)	0.94		
History of asthma or atopy	1.33 (0.96–2.8)	0.73		
VDR *Fok* I genotype (FF vs Ff + ff)	4.05 (1.9–8.5)	<0.001	4.3 (2.6–9.2)	<0.001
VDR allele (F vs f)	1.45 (1.0–1.9)	0.01	2.25 (1.1–3.7)	0.003
Serum 25(OH) vitamin D (<20 ng/mL)	2.08 (1.82–4.3)	0.04	2.6 (1.96–4.9)	0.002

*COVID-19* coronavirus disease 2019, *VDR* vitamin D receptor.

*P* value < 0.05 indicates a significant difference.

^a^Adjusted for age, sex, season at enrollment, and household crowding.

## Discussion

A high prevalence of vitamin D deficiency has been reported in pediatric and adolescent population across the globe. It has been postulated that vitamin D deficiency is a risk factor for the epidemic of LRTIs in Chinese, Canadian, and Egyptian cohorts.^24–26^ In our study, patients diagnosed with COVID-19 had significantly lower vitamin D serum levels compared to the control group. Moreover, vitamin D deficiency and insufficiency was detected in more than half (52%) of COVID-19 patients; although our study population resides in Delta and Upper Egypt, both regions have abundant sunlight exposure throughout the year. Of note, the distribution of COVID-19 severity according to 25(OH) D levels was not found significantly different between the studied groups. Our findings confirm and extend recently published data in pediatric and adult age groups.^17–19,27–29^

Recently, two studies investigated vitamin D deficiency as a risk factor for COVID-19 in Turkish children and adolescents. Yılmaz and Şen^17^ reported that 72.5% of their cases were vitamin D deficient and patients admitted to ICU had vitamin D level of <10 ng/mL. Consistent with our findings, the authors concluded that vitamin D deficiency may be associated with increased susceptibility to COVID‐19 but not disease severity in pediatric population. A similar study by Alpcan et al.^18^ reported that vitamin D deficiency was a risk factor for the development of ARD and may be correlated to COVID-19 severity among Turkish children.

Akoğlu et al.^19^ reported that patients with moderate COVID-19 severity had lower 25(OH) D as compared to the mild disease group. The authors added that vitamin D deficiency may worsen the aggravation of pulmonary involvement by SARS-COV-2. Beyazgül et al.^30^ reported that school-aged children and adolescents had low 25 (OH) D levels during COVID-19 pandemic period due to the restriction rules applied to prevent COVID-19 spreads. Darren et al.^31^ was the first to study vitamin D status in pediatric multi-system inflammatory syndrome associated with SARS-CoV-2 (PIMS-TS). They reported that 72% of their cohort was vitamin D deficient and specifically all PICU patients had suboptimal vitamin D level. The authors suggested that vitamin D deficiency could be a modifiable risk factor for PIMS-TS because of its immune-modulatory action on inflammatory cytokine signaling.

The largest meta-analysis that involved more than one million adult individuals suggested a potential link between vitamin D status and susceptibility to SARS-CoV-2 infection. They added that sufficient vitamin D levels may decrease the risk of becoming infected by SARS-CoV-2.^27^ An Israeli epidemiological study reported that 25(OH) D levels <20 ng/mL almost doubled the risk for SARS-CoV-2 infection and hospitalization.^28^ Pinzon et al. reported a 90% prevalence of vitamin D deficiency among COVID-19 patients in Indonesia although it is a tropical country with a plenty of sunny weather.^29^ A similar study in Italy reported vitamin D deficiency in 81% of patients admitted to ICU with ARF due to COVID-19. The authors added that severe vitamin D deficiency may be a marker of poor prognosis in these patients.^32^ Whether any link exists between vitamin D deficiency and the severity of COVID-19 requires further evidence.

In keeping with a meta-analysis performed by de Souza et al.,^33^ the most prevalent symptoms among studied cohort were fever, dry cough, and shortness of breath, followed by diarrhea, vomiting, and fatigue. Moreover, dry cough and development of pneumonia were more frequent in patients who had deficient level of vitamin D (Group 1) than those with normal vitamin D status (Group 2) although it does not reach a statistical significance. Among the most common abnormal laboratory findings were lymphopenia, elevated CRP, and D-dimer level. However, no significant difference was evident between both groups in terms of measured laboratory parameters and inflammatory biomarkers. Fortunately, all patients survived so we could not evaluate the association between vitamin D levels and COVID-19 mortality.

In animal models, severe acute lung injury was accompanied by an increase in pulmonary renin and angiotensin II levels and excessive induction of angiopoietin (Ang)-2 and myosin light chain (MLC). The vitamin D-VDR signaling protects the pulmonary vascular barrier and prevents acute lung injury by targeting the renin–angiotensin cascade and blocking the Ang-2-Tie-2-MLC kinase pathway.^34^

It has been shown that 1,25-OH2-vit D exhibit antiviral inhibitory effect on human nasal epithelial cells infected with SARS-CoV-2 virus.^35^ SARS-CoV-2 enters host cells after its protein S (Spike) binds to ACE2 receptors. The primary targets of SARS-CoV-2 are alveolar cell type-II on which ACE2 receptors is highly expressed.^3^ Vitamin D agonist calcitriol enhances the expression of ACE2 and increases soluble ACE2 (sACE2), which may be responsible for trapping and inactivating the virus. Calcitriol also suppresses renin expression and serves as a negative regulator of renin–angiotensin–aldosterone (RAS) system, which is exacerbated in SARS-CoV-2 infection, making more angiotensin II (Ang-II) available to cause tissue damage, inflammation, and multi-organ failure.^36^

Vitamin D modulates adaptive immune response as it was found to downregulate the inflammatory cytokine expression, in a VDR-dependent manner, from a Th1 to a Th2 profile. It also inhibits the development of T helper 17 cells and enhances T regulatory (T reg) lymphocytes, thus mitigating tissue damage and inflammation.^37^ Moreover, it suppresses the expression of pro-inflammatory cytokines, specifically interleukin-6 and tumor necrosis factor-α (TNF-α) as both are predictors of severe COVID-19 and worse clinical outcome. Sufficient vitamin D status may help to blunt or dampen the cytokine storm by simultaneously boosting the innate immune response and reducing the overactivation of the adaptive immunity.^11^ A recent multicenter study by Shafiek et al. investigated serum cytokine profile in pediatric patients diagnosed with COVID-19 pneumonia. They reported markedly elevated serum pro-inflammatory cytokines and chemokines indicating a cytokine storm following SARS-CoV-2 infection.^38^ However, a preliminary data on vitamin D status and concomitant cytokine profile in pediatric COVID-19 patients is still lacking.

The VDR polymorphisms have been reported to be associated with susceptibility to lower respiratory infections including respiratory syncytial virus bronchiolitis,^39^ symptomatic pertussis,^40^ and community-acquired pneumonia^41^ in South African, Dutch, and Chinese Han children, respectively. To our knowledge, ours is the first such study to investigate the VDR *Fok* I polymorphism in Caucasian children and adolescents with COVID-19.

In our study, the VDR *Fok* I *homozygous FF* genotype and *F* allele were significantly more represented in COVID-19 patients as compared to the control group. Patients carrying the *FF* genotype had 4.05-fold increased susceptibility to COVID-19. By contrast, the *Fok* I *Ff* genotype showed a significant negative association with COVID-19 risk suggesting that the VDR *Fok* I *f* allele may confer protection against SARS-CoV-2 infection. In an attempt to explain our findings, we investigated the 25(OH) D serum level in relation to the studied VDR polymorphism, which was found to be significantly lower in patients homozygous for the *Fok* I *FF* genotype compared to those carrying the *ff* and *Ff* genotypes. These results are concordant with a recent multicenter study by Abouzeid et al.^42^ who investigated the VDR *Fok* I polymorphism on genomic DNAs of 300 children diagnosed with community-acquired pneumonia. The authors reported that the VDR *Fok* I FF genotype was associated with lower serum 25(OH) D levels and may confer susceptibility to CAP and related hospital mortality in the under-five Egyptian children.

In the current study, no significant association was evident between the VDR FokI genotype distribution and COVID-19 severity or clinical outcome. Of note, our logistic regression model revealed that vitamin D deficiency and the VDR *Fok* I FF genotype were independent risk factors for COVID-19 among the studied patients controlling for age, sex, season at enrollment, and household crowding as potential confounders. On the contrary, Apaydin et al. reported that the FokI genotypes were similarly distributed in the COVID-19 and control groups. They also found that the Ff genotype for Fok I was associated with disease severity (OR: 3.17) in Turkish population.^43^ A similar study reported that FokI genotypic distributions exhibited a remarkable discrepancy in adult patients with severe COVID-19 compared to asymptomatic cases. The authors concluded that the *Fok*I polymorphism may represent a pinpointed associated factor with severity and outcome of COVID-19 in the Iranian population.^44^

1,25-OH2-vit D regulates its own serum levels and its precursor 25(OH) D by a VDR-dependent negative feedback loop. Unlike other VDR SNP, the FokI polymorphism at the start codon results in two VDR isoforms with different structure. The *f* allele encodes a longer and less active VDR protein than that translated by the *F* allele. Consequently, the *f* VDR isoform allows more synthesis of 25(OH) D, which may partially explain the observed higher vitamin D serum levels in FokI homozygous *ff* genotype.^16,45^ Together with our findings, it is plausible to speculate that the VDR *Fok* I FF genotype, being associated with lower serum 25(OH) D levels, may constitute an independent risk factor for susceptibility to COVID-19.

As evidence on the link between vitamin D status and COVID-19 in pediatric population continues to emerge, clinical trials should be urgently conducted to test for causality and to evaluate the efficacy of vitamin D supplementation for prophylaxis and treatment of COVID-19.

However, several limitations should be considered in this study. First, the small sample size may necessitate adopting a genome-wide association study across various ethnic populations. Second, we have studied *Fok* I *SNP* in the VDR gene that may represent linkage disequilibrium with other VDR genomic loci, but this is yet to be defined. Third, we have measured 25(OH) D levels at SARS-CoV-2 diagnosis (at the initial phase of illness), so the possibility of reverse causality cannot be completely ruled out. Finally, there is a lack of sufficient data about many environmental risk factors that may predispose to acute respiratory infections including COVID-19 in a genetically susceptible child.

## In conclusion

Vitamin D deficiency and the VDR *Fok* I polymorphism may constitute independent risk factors for susceptibility to COVID-19 in Egyptian children and adolescents.

Finally, the potential role of vitamin D in pathophysiology of COVID-19 should be further addressed in large-scale studies taking into account the VDR polymorphisms.

## Data Availability

All data generated during and/or analyzed during the current study are available from the corresponding author on reasonable request.

## References

[1] World Health Organization. WHO coronavirus (COVID-19) dashboard. https://covid19.who.int (2022).

[2] Huang C (2020). Clinical features of patients infected with 2019 novel coronavirus in Wuhan, China. Lancet.

[3] Hoffmann M (2020). SARS-CoV-2 cell entry depends on ACE2 and TMPRSS2 and is blocked by a clinically proven protease inhibitor. Cell.

[4] Guan WJ (2020). Clinical characteristics of coronavirus disease 2019 in China. N. Engl. J. Med..

[5] Wang D (2020). Clinical characteristics of 138 hospitalized patients with 2019 novel coronavirus-infected pneumonia in Wuhan, China. JAMA.

[6] Pike JW, Meyer MB (2010). The vitamin D receptor: new paradigms for the regulation of gene expression by 1,25-dihydroxyvitamin D(3). Endocrinol. Metab. Clin. North Am..

[7] Martineau, A. R. et al. Vitamin D supplementation to prevent acute respiratory tract infections: systematic review and meta-analysis of individual participant data. *BMJ* **356**, i6583 (2017). 10.1136/bmj.i6583 PMC5310969 28202713

[8] Dancer RC (2015). Vitamin D deficiency contributes directly to the acute respiratory distress syndrome (ARDS). Thorax.

[9] Underwood MA, Bevins CL (2010). Defensin-barbed innate immunity: clinical associations in the pediatric population. Pediatrics.

[10] Long QX (2020). Clinical and immunological assessment of asymptomatic SARS-CoV-2 infections. Nat. Med..

[11] Grant WB (2020). Evidence that vitamin D supplementation could reduce risk of influenza and COVID-19 infections and deaths. Nutrients.

[12] Li, Y., Li, Q., Zhang, N. & Liu, Z. Sunlight and vitamin D in the prevention of coronavirus disease (COVID-19) infection and mortality in the United States. Preprint at 10.21203/rs.3.rs-32499/v1 (2020).

[13] Daneshkhah A (2020). Evidence for possible association of vitamin D status with cytokine storm and unregulated inflammation in COVID-19 patients. Aging Clin. Exp. Res..

[14] Wang Q, Xi B, Reilly KH, Liu M, Fu M (2012). Quantitative assessment of the associations between four polymorphisms (FokI, ApaI, BsmI, TaqI) of vitamin D receptor gene and risk of diabetes mellitus. Mol. Biol. Rep..

[15] Neill O (2013). Vitamin D receptor gene expression and function in a South African population: ethnicity, vitamin D and FokI. PLoS ONE.

[16] van Etten E (2007). The vitamin D receptor gene FokI polymorphism: functional impact on the immune system. Eur. J. Immunol..

[17] Yılmaz K, Şen V (2020). Is vitamin D deficiency a risk factor for COVID-19 in children?. Pediatr. Pulmonol..

[18] Alpcan A, Tursun S, Kandur Y (2021). Vitamin D levels in children with COVID-19: a report from Turkey. Epidemiol. Infect..

[19] Akoğlu HA (2021). Evaluation of childhood COVID-19 cases: a retrospective analysis. J. Pediatr. Infect. Dis..

[20] Chen ZM (2020). Diagnosis and treatment recommendations for pediatric respiratory infection caused by the 2019 novel coronavirus. World J. Pediatr..

[21] Holick MF (2017). The vitamin D deficiency pandemic: approaches for diagnosis, treatment and prevention. Rev. Endocr. Metab. Disord..

[22] Freire-Paspuel B (2021). Analytical and clinical comparison of Viasure (CerTest Biotec) and 2019-nCoV CDC (IDT) RT-qPCR kits for SARS-CoV2 diagnosis. Virology.

[23] Fedirko V (2012). Pre-diagnostic 25-hydroxyvitamin D, VDR and CASR polymorphisms and survival in patients with colorectal cancer in Western European Populations. Cancer Epidemiol. Biomark. Prev..

[24] Li W (2018). Association between serum 25-hydroxyvitamin D concentration and pulmonary infection in children. Medicine.

[25] McNally JD (2009). Vitamin D deficiency in young children with severe acute lower respiratory infection. Pediatr. Pulmonol..

[26] El Basha N, Mohsen M, Kamal M, Mehaney D (2014). Association of vitamin D deficiency with severe pneumonia in hospitalized children under 5 years. Comp. Clin. Pathol..

[27] Chiodini I (2021). Vitamin D status and SARS-CoV-2 infection and COVID-19 clinical outcomes. Front Public Health.

[28] Merzon E (2020). Low plasma 25(OH) vitamin D level is associated with increased risk of COVID-19 infection: an Israeli population-based study. FEBS J..

[29] Pinzon RT, Pradana AW (2020). Vitamin D deficiency among patients with COVID-19: case series and recent literature review. Trop. Med. Health.

[30] Beyazgül G (2022). How vitamin D levels of children changed during COVID-19 pandemic: a comparison of pre-pandemic and pandemic periods. J. Clin. Res. Pediatr. Endocrinol..

[31] Darren A (2022). Vitamin D status of children with paediatric inflammatory multisystem syndrome temporally associated with severe acute respiratory syndrome coronavirus 2 (PIMS-TS). Br. J. Nutr..

[32] Carpagnano GE (2020). Vitamin D deficiency as a predictor of poor prognosis in patients with acute respiratory failure due to COVID-19. J. Endocrinol. Investig..

[33] de Souza TH, Nadal JA, Nogueira RJN, Pereira RM, Brandão MB (2020). Clinical manifestations of children with COVID-19: a systematic review. Pediatr. Pulmonol..

[34] Kong J (2013). VDR attenuates acute lung injury by blocking Ang-2-Tie-2 pathway and renin-angiotensin system. Mol. Endocrinol..

[35] Greiller CL, Martineau AR (2015). Modulation of the immune response to respiratory viruses by vitamin D. Nutrients.

[36] Yuan W (2007). 1, 25-dihydroxyvitamin D3 suppresses renin gene transcription by blocking the activity of the cyclic AMP response element in the renin gene promoter. J. Biol. Chem..

[37] Overbergh L (2000). 1alpha,25-dihydroxyvitamin D3 induces an autoantigen-specific T-helper 1/T-helper 2 immune shift in NOD mice immunized with GAD65 (p524-543). Diabetes.

[38] Shafiek HK (2021). Cytokine profile in Egyptian children and adolescents with COVID-19 pneumonia: a multicenter study. Pediatr. Pulmonol..

[39] Kresfelder TL, Janssen R, Bont L, Pretorius M, Venter M (2011). Confirmation of an association between single nucleotide polymorphisms in the VDR gene with respiratory syncytial virus related disease in South African children. J. Med. Virol..

[40] Han WG (2016). Association of vitamin D receptor polymorphism with susceptibility to symptomatic pertussis. PLoS ONE.

[41] Li W (2015). Polymorphism rs2239185 in vitamin D receptor gene is associated with severe community-acquired pneumonia of children in Chinese Han population: a case–control study. Eur. J. Pediatr..

[42] Abouzeid H (2018). Association of vitamin D receptor gene FokI polymorphism and susceptibility to CAP in Egyptian children: a multicenter study. Pediatr. Res..

[43] Apaydin T (2022). Effects of vitamin D receptor gene polymorphisms on the prognosis of COVID-19. Clin. Endocrinol..

[44] Abdollahzadeh R (2021). Association of Vitamin D receptor gene polymorphisms and clinical/severe outcomes of COVID-19 patients. Infect. Genet. Evol..

[45] Jurutka PW (2000). The polymorphic N terminus in human vitamin D receptor isoforms influences transcriptional activity by modulating interaction with transcription factor IIB. Mol. Endocrinol..

